# Environmental exposure to rare earth elements and respiratory health: an emerging public health concern

**DOI:** 10.3389/fpubh.2026.1811406

**Published:** 2026-04-15

**Authors:** Jie Zhou, Pengcheng Liang, Weijian Zhu, Runan Zhang, Bin Zhong

**Affiliations:** 1 School of the First Clinical Medicine, Gannan Medical University, Ganzhou, China; 2 School of Pharmacy, Gannan Medical University, Ganzhou, China; 3 Chongyi County People’s Hospital, Ganzhou, China; 4 Department of Respiratory Medicine, The First Affiliated Hospital of Gannan Medical University, Ganzhou, China

**Keywords:** calcium homeostasis, emerging contaminant, inflammation, inhalation exposure, oxidative stress, rare earth element, respiratory toxicity

## Abstract

Rare earth elements (REEs) are increasingly released into the environment due to intensive mining, industrial processing, and expanding technological applications, resulting in widespread human exposure. Within the respiratory exposome framework, REEs have increasingly been recognized as a potentially important class of airborne contaminants. Fine and ultrafine REE-containing particles can penetrate deeply into the distal lung, where they exhibit high biopersistence and limited clearance. Epidemiological evidence from mining and industrial regions suggests that elevated internal REE burdens may be associated with increased prevalence of respiratory symptoms and chronic lung diseases, including bronchitis and interstitial lung disease. Toxicokinetic and experimental studies provide mechanistic support, demonstrating that inhaled REEs preferentially deposit in the alveolar region, interact with epithelial and immune cells, and may translocate into systemic circulation. At the molecular level, REEs have been shown to induce oxidative stress, immune and inflammatory dysregulation, and calcium homeostasis imbalance in experimental models, thereby promoting tissue injury and remodeling. These processes may contribute to a progressive pathological continuum from persistent inflammation to fibrosis and, potentially, tumorigenesis. Notably, exposure characteristics—including particle physicochemical properties, dose, co-exposure scenarios, and host susceptibility—critically shape health outcomes in real-world settings. Despite accumulating evidence, key uncertainties remain regarding human-relevant exposure thresholds, long-term dose–response relationships, and validated biomarkers of effect. Current knowledge is still largely derived from experimental models, with limited integration into population-based risk assessment. Overall, this review uses a structured literature search and narrative synthesis approach to integrate environmental exposure pathways, toxicokinetic characteristics, and mechanistic evidence within an exposome-oriented framework. It highlights that REEs represent emerging inhalation hazards with the potential to contribute to the burden of chronic respiratory diseases, underscoring the need for improved exposure assessment, biomonitoring strategies, and evidence-based public health interventions.

## Graphical abstract

**Figure:**
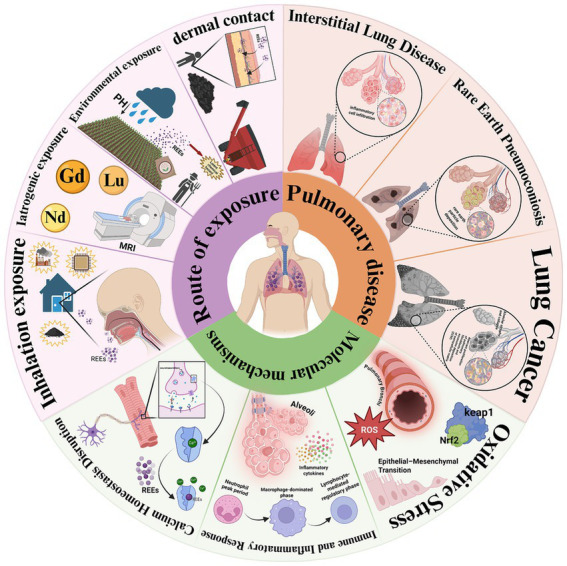
Created with BioRender.com.

## Highlights

- Rare earth elements are emerging airborne contaminants that expand the respiratory exposome in mining and industrial regions.
- Persistent pulmonary deposition and limited clearance provide a toxicokinetic basis for chronic respiratory injury.
- Convergent pathways involving oxidative stress, immune dysregulation, and calcium imbalance support a progression toward fibrosis.
- Integration of environmental monitoring, biomonitoring, and mechanistic evidence is urgently needed to inform risk assessment and public health policy.

## 1 Introduction

Rare earth elements (REEs) comprise a group of 17 metallic elements, including the 15 lanthanides as well as yttrium (Y) and scandium (Sc). Based on atomic number and physicochemical properties, REEs are typically classified into two categories: light rare earth elements (LREEs), which include lanthanum (La), cerium (Ce), praseodymium (Pr), neodymium (Nd), promethium (Pm), samarium (Sm), and europium (Eu); and heavy rare earth elements (HREEs), which include gadolinium (Gd), terbium (Tb), dysprosium (Dy), holmium (Ho), erbium (Er), thulium (Tm), ytterbium (Yb), lutetium (Lu), scandium (Sc), and yttrium (Y) (1, 2). To date, China remains the largest global supplier and holds the most abundant reserves of REEs, especially in southern Jiangxi, which is known as the “Kingdom of Rare Earths” (3–5). Owing to their unique electronic structures and physicochemical properties, REEs play an irreplaceable role in modern industry and advanced technologies. REEs are widely utilized in diverse fields, including consumer electronics (e.g., smartphones, electric vehicles), aerospace, renewable energy technologies, medical imaging (e.g., MRI contrast agents), and agricultural feed additives (6). However, given their extensive industrial and technological applications (7–9), rare earth elements (REEs) are increasingly released into the environment through mining, manufacturing, and waste disposal. Although not yet strictly regulated, their persistence, bioaccumulation potential, and emerging evidence of toxicity have raised growing concern. These features position REEs as a new class of emerging contaminants, representing chemicals of increasing environmental concern whose health and ecological risks are only beginning to be understood (10–12). The migration and enrichment of REEs in the environment result in human exposure through multiple pathways, including the respiratory tract, gastrointestinal tract, and skin, thereby posing potential health risks (13, 14). Among these, inhalation exposure via the respiratory tract is the most common route (15). Moreover, several studies have reported that REE particles exhibit strong biopersistence, remaining in lung tissue for prolonged periods (16–19), thereby inducing chronic injury and even irreversible structural alterations (20, 21). The respiratory system, especially the lung, as the primary interface with the external environment, not only performs defensive functions but may also serve as a “gateway” for the protection of human health.

In the context of increasing global emphasis on green development and ecological protection, scientifically assessing the health impacts of REEs, especially on the lung—particularly among occupational populations and environmentally susceptible groups such as children and the older adult(s)—has become a critical issue in public health research. However, the kinetic behavior, tissue distribution, and clearance mechanisms of inhaled REEs within the lung remain incompletely understood. A structured literature search strategy was employed to ensure comprehensive and balanced coverage of the available evidence. Therefore, the main purpose of this review is to focus on the current literature to provide an overview and discuss the hazardous effects of REE exposure on the lung.

## 2 Literature search strategy and selection criteria

This review was conducted as a structured narrative synthesis supported by a transparent and reproducible literature search. PubMed and Web of Science were used as the primary databases, with Scopus employed as a supplementary source to enhance coverage and minimize the risk of missing relevant studies.

### 2.1 Search strategy

A comprehensive search strategy was developed using a combination of controlled vocabulary (Medical Subject Headings, MeSH) and free-text terms.

The PubMed search strategy was as follows:

(“Rare Earth Elements” OR “rare earth element” OR REEs OR lanthanide OR cerium OR lanthanum OR neodymium OR yttrium OR gadolinium OR dysprosium) AND (“Respiratory Tract” OR lung OR pulmonary OR respiratory) AND (“Inhalation Exposure” OR inhalation OR aerosol OR particulate matter OR dust OR nanoparticle) AND (toxicity OR toxic OR inflammation OR fibrosis OR oxidative stress OR lung disease)

This search yielded 477 records.

The Web of Science Core Collection was searched using the following query:

TS = ((“rare earth element” OR REEs OR lanthanide OR cerium OR lanthanum OR neodymium OR yttrium OR gadolinium OR dysprosium) AND (lung OR pulmonary OR respiratory) AND (inhalation OR aerosol OR “particulate matter” OR dust OR nanoparticle) AND (toxic OR inflammation OR fibrosis OR “oxidative stress” OR “lung disease”))

This search yielded 135 records.

In addition, Scopus was searched using similar keyword combinations as a supplementary database to ensure comprehensive coverage.

The final search was conducted up to March 29, 2026, without restrictions on study design.

### 2.2 Study selection

All retrieved records were imported into Endnote, and duplicates were removed prior to screening. Titles and abstracts were screened for relevance, followed by full-text assessment of potentially eligible studies.

Studies were included if they: (1) investigated exposure to rare earth elements, particularly via inhalation; (2) reported respiratory-related outcomes, including epidemiological, toxicological, or mechanistic findings; (3) provided relevant insights into toxicokinetics or biological effects in the respiratory system.

Studies were excluded if they: (1) were not related to respiratory health; (2) focused solely on non-respiratory exposure routes without mechanistic relevance; (3) lacked sufficient methodological detail.

After applying these criteria, 141 studies were included in the final synthesis. Reference lists of relevant articles were also manually screened to identify additional eligible studies.

### 2.3 Synthesis approach

Given the heterogeneity in study design, exposure characteristics, and outcome measures, a qualitative narrative synthesis approach was adopted rather than a formal systematic review or meta-analysis.

## 3 Rare earth element exposure

### 3.1 Rare earth element exposure pathways

The migration and enrichment of REEs in the environment result in human exposure through multiple pathways, including iatrogenic exposure, environmental exposure, and dermal contact, among others, thereby posing potential health risks (13, 14). Iatrogenic exposure primarily involves the use of gadolinium (Gd)-based contrast agents in medical procedures such as magnetic resonance imaging (MRI) (22). Although the main concern has focused on toxicity in other organs, their metabolic and distribution patterns suggest potential impacts on the lung. Environmental exposure is also noteworthy. Columnar simulation experiments have shown that decreased pH in acid rain significantly enhances the mobility of REEs in soil, with leaching amounts inversely correlated with acidity. Agricultural activities, such as ammonium fertilizer application, can further activate fixed-state REEs via ion-exchange reactions, resulting in widespread dispersion. Anthropogenic activities including mining and agriculture may contribute to the large-scale release of REEs from soil reservoirs into the environment, forming a “Latent chemical hazard” (23), which may lead to inhalable exposure via dust and aerosols. Dermal contact should not be overlooked; studies in mining occupational populations indicate that prolonged skin contact can facilitate percutaneous absorption of REEs (24), which may subsequently enter systemic circulation and migrate to the lungs or other organs, indirectly contributing to or exacerbating lung toxicity.

**Figure 1:**
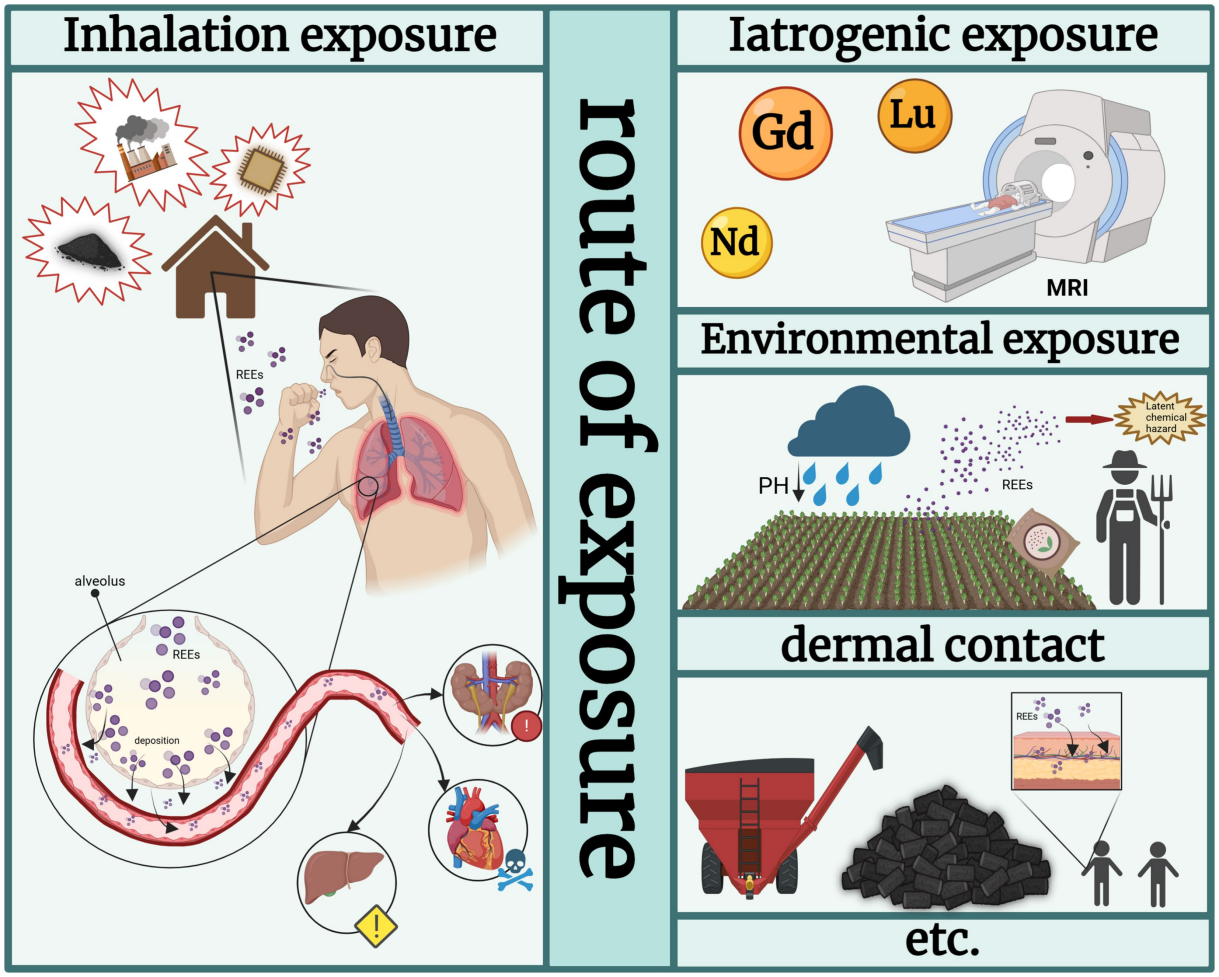
Rare earth element exposure. Created with BioRender.com.

However, inhalation is the primary route of exposure to airborne particulate matter (15) (see Figure 1). Respiratory intake of REE dust or particles represents the main form of exposure to the lung, particularly in regions with REE mining, smelting, or concentrated manufacturing facilities (25). Particulates generated during REE extraction and processing, especially nano- or submicron-sized rare earth oxides (e.g., CeO₂, La₂O₃, Y₂O₃), can be carried into the human respiratory tract via aerosols and deposit in the alveolar region (18). PM2.5 particles, defined as particulate matter with an aerodynamic diameter≤2.5 μm, are small enough to penetrate the deepest regions of the human respiratory tract (26). For example, an assessment conducted in Baotou, the largest REE processing city in northern China, measured PM2.5 concentrations in different areas and found REE levels of 56.9 ng/m^3^ in August 2013 and 15.3 ng/m^3^ in April 2014, significantly higher than those in non-REE mining areas. The estimated daily intake of REEs via PM2.5 inhalation among the local population ranged from 5.09 × 10^−7^ to 2.25 × 10^−5^ mg/kg/day (27). Deposited REEs have been reported to penetrate the alveolar epithelium, cross the alveolar–capillary barrier, and enter systemic circulation, ultimately reaching extrapulmonary organs such as the heart, liver, and kidneys (1, 28). Epidemiological studies support these findings; in a southern Chinese REE mining region, residents living near the mines, particularly children, exhibited significantly elevated REE levels in hair samples, which were associated with increased incidence of respiratory symptoms, including chronic cough, asthma, and bronchitis (29–33). However, it should be noted that many of these epidemiological studies are conducted in complex environmental and occupational settings, where potential confounding factors—such as smoking status, age, socioeconomic status, and co-exposure to other airborne pollutants or metals—may not be fully controlled. Therefore, the observed associations should be interpreted with caution. What is more, the inhalation toxicity of REEs remains a matter of debate. Except for CeO₂, quantified human-relevant inhalation exposure data are scarce, and no reference inhalation doses have been established for lanthanides (34).

Overall, available evidence suggests that REEs may exert toxic effects on the lung, with inhalation representing the most significant and direct exposure route, although the strength of evidence varies across study types. Nevertheless, less frequent indirect pathways also warrant attention, providing theoretical guidance for defining protective thresholds and implementing preventive measures for susceptible populations and public health authorities.

### 3.2 REE exposure in the context of the respiratory exposome

The concept of the exposome provides a comprehensive framework for understanding the health effects of environmental exposures across the lifespan. Within this framework, REE exposure should not be considered in isolation but rather as part of a complex mixture of environmental stressors.

First, individuals are typically exposed to REEs in combination with other airborne pollutants, such as fine particulate matter (PM₂.₅), heavy metals, and industrial emissions. These co-exposures may result in additive, synergistic, or antagonistic effects, potentially modifying the respiratory toxicity of REEs. However, current studies largely focus on single-agent exposures, limiting our understanding of real-world exposure scenarios.

Second, the exposome emphasizes the importance of life-course exposure assessment. Exposure to REEs may occur at multiple stages of life, including occupational exposure in adulthood and environmental exposure beginning in early life. The timing, duration, and cumulative burden of exposure may influence susceptibility to respiratory diseases, yet longitudinal data in this area remain scarce.

Third, interindividual variability in response to REE exposure may be influenced by genetic susceptibility and host factors. Differences in antioxidant capacity, inflammatory responses, and xenobiotic metabolism could modulate the biological effects of REEs. Integrating genetic and molecular data with environmental exposure assessment represents an important direction for future research.

Overall, adopting an exposome-based perspective may improve the interpretation of existing evidence and facilitate a more holistic understanding of REE-associated respiratory health risks.

## 4 Toxicokinetic of rare earth elements

Importantly, the toxicokinetic behavior of rare earth elements (REEs) is strongly dependent on their physicochemical form, particularly the distinction between poorly soluble particulate forms (e.g., rare earth oxides and nanoparticles) and soluble ionic forms (e.g., chlorides and other salts). These forms differ substantially in their absorption, distribution, biopersistence, and clearance. Insoluble particles tend to deposit in the respiratory tract and exhibit prolonged pulmonary retention, whereas soluble REE salts are more likely to dissolve, release free ions, and undergo systemic distribution. Therefore, distinguishing between these forms is essential for accurately interpreting toxicological outcomes and underlying mechanisms.

The toxicokinetic of REEs begin with absorption. REEs are most absorbed following inhalation, typically in the form of particulate matter or nanoparticles (15). Upon entry into the respiratory tract, they first encounter the primary defenses of the immune system (35). Larger particles (>2.5 μm) tend to deposit in the upper respiratory tract and are subsequently cleared through the mucociliary escalator. Fine particles (e.g., PM2.5) can reach the terminal bronchioles and alveoli, whereas nanoparticles (<100 nm), such as CeO₂ and La₂O₃, are readily taken up by alveolar epithelial cells and macrophages (36). A small fraction of these nanoparticles is even capable of penetrating the air–blood barrier and entering systemic circulation. Additional absorption pathways have also been described, including macrophage-mediated phagocytosis, trans-epithelial transport, and intracellular endocytosis–lysosomal trafficking of nanoparticles (37). Importantly, the extent of absorption is strongly influenced by particle size, morphology, solubility, and surface charge.

Following absorption, REEs exhibit characteristic patterns of distribution. Current evidence indicates that inhaled REEs predominantly accumulate in the lungs, forming the pathological basis of REE pneumoconiosis observed in occupationally exposed workers (38). Studies have suggested that REE particles entering the human body can induce localized oxidative stress, inflammatory cell infiltration, bronchial epithelial injury, and impaired alveolar gas exchange (39–41). Animal experiments further support that short-term or repeated exposure to REE nanoparticles may causes structural damage to lung tissue, characterized by bronchial epithelial shedding, alveolar inflammation, and a tendency toward interstitial pulmonary fibrosis (40, 42). Notably, only under conditions involving nanoscale particles, high exposure levels, or compromised biological barriers have trace amounts of REEs been reported in the central nervous system (43).

Regarding metabolic transformation, REEs, being metallic elements, do not undergo classical biotransformation as organic compounds do. Instead, they participate in various physicochemical conversion processes. Three major transformation pathways have been identified: (1) dissolution–reprecipitation, such as the Ce^3+^/Ce^4+^ redox cycling of CeO₂ nanoparticles in inflammatory microenvironments, followed by precipitation with phosphate to form insoluble deposits (44); (2) protein binding and alterations in surface coatings, wherein REEs interact with biomolecules in biological fluids to form a “protein corona” that modulates their biological and toxicological behavior (45); (3) redox modification of nanoparticle surfaces, exemplified by the “self-regenerating” redox cycling of CeO₂, which underlies its dual capacity to generate or scavenge reactive oxygen species (46).

Finally, REE excretion is generally slow. The primary elimination routes are fecal excretion, transpulmonary clearance, and urinary excretion (47). Overall, the toxicokinetic characteristics of REEs suggest pronounced pulmonary retention and limited clearance. Notably, nano-sized REE particles may penetrate the alveolar epithelial barrier and enter the systemic circulation, distributing to distant organs such as the liver, spleen, and kidneys, with smaller amounts detected in bone and lymph nodes, thereby increasing the risk of systemic toxicity.

However, the kinetic behavior, tissue distribution, and clearance mechanisms of inhaled REEs within the respiratory tract remain incompletely understood. In particular, the health effects under conditions of long-term, low-dose, or mixed exposures require systematic investigation (1, 48, 49).

## 5 REE-associated pulmonary diseases

Based on the toxicokinetic characteristics of rare earth elements, it is evident that they preferentially deposit in the lungs and exhibit pronounced pulmonary retention. In recent years, with the extensive application of rare earth elements (REEs) in industry and medicine, their lung toxicity has become increasingly evident. Current evidence suggests that REE particles can persist in the lungs for prolonged periods (17), triggering inflammation, fibrosis, and even tumorigenesis (see Table 1).

**Table 1:** A summary of rare earth elements that primarily cause lung damage.

Element	Deposition sites	Toxic effects	Reference
Y	Bronchial/alveolar regions, macrophages	chronic inflammatory	(15)
La	Bronchial epithelium, bronchioles, alveolar space, macrophages	Inflammatory cell infiltration, oxidative stress, persistent macrophage retention, early fibrotic responses	(138)
Ce	Alveolar surface, macrophages, interstitium	Pronounced redox cycling (Ce^3+^↔Ce^4+^), ROS modulation, inflammation, cell death, fibrosis; dose- and valence-dependent biphasic effects	(58)
Sm	Alveoli, macrophages, bronchial epithelium	Inflammation, cytotoxicity	(139)
Gd	Alveoli, bronchial epithelium, interstitium;	Inflammation, potential fibrotic risk;	(140)
Dy	Interstitium, macrophages, epithelial surfaces	Oxidative stress, inflammation, extracellular matrix remodeling	(141)

Y, yttrium; La, lanthanum; Ce, cerium; Sm, samarium; Gd, gadolinium; Dy, dysprosium.

**Figure 2:**
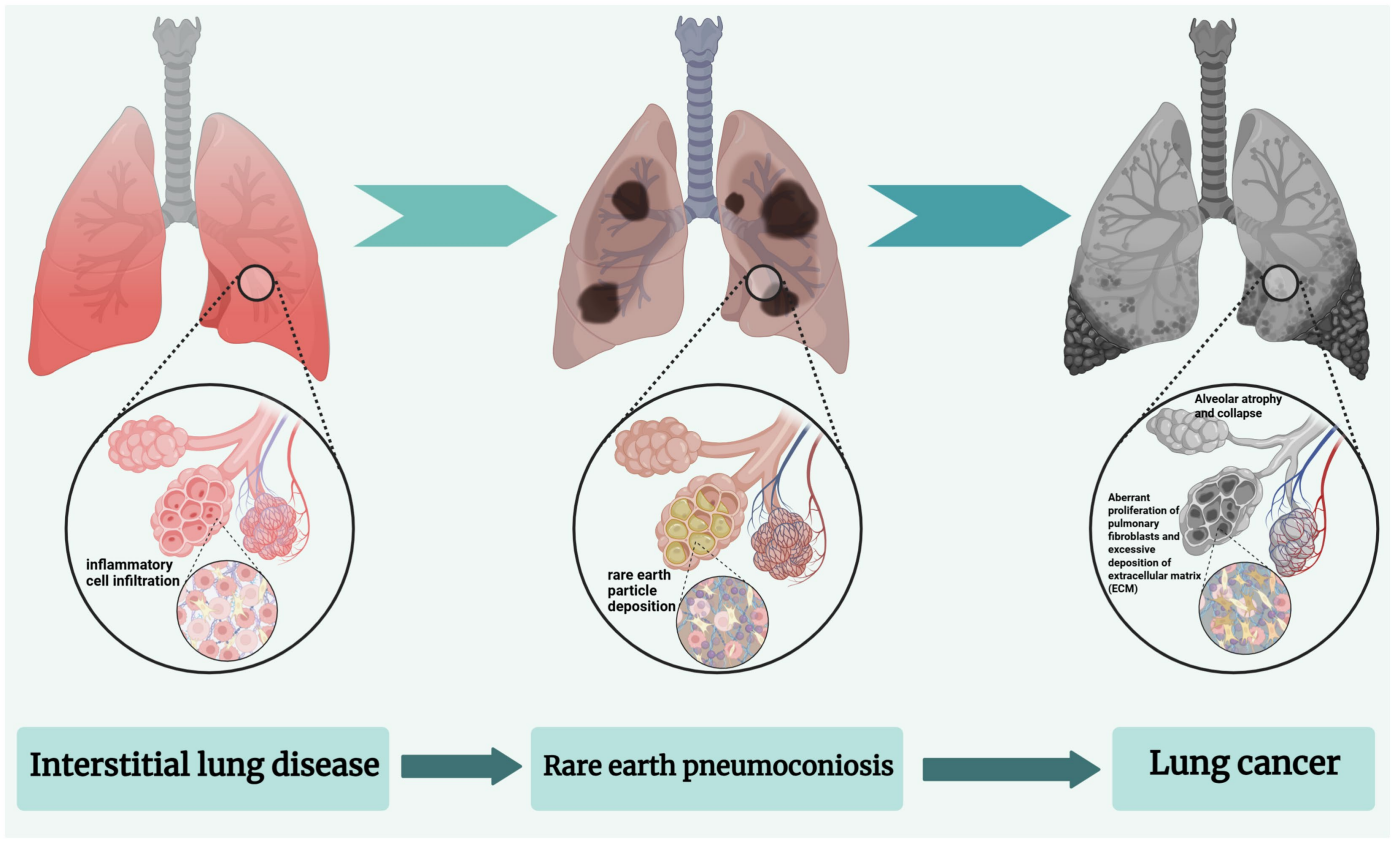
The continuous pathological spectrum of inflammation–fibrosis–tumor. Created with BioRender.com.

Therefore, this forms a continuous pathological spectrum of “inflammation–fibrosis–tumor” (50). Specifically, interstitial pneumonia involves oxidative stress and inflammation mediated by multiple signaling pathways (51, 52); pneumoconiosis caused by REEs is mainly characterized by particle deposition and chronic fibrosis (17); while the risk of lung cancer may be associated with DNA damage, gene mutations, and microenvironmental remodeling (53) (see Figure 2). The following sections review recent progress on interstitial pneumonia, REE-related pneumoconiosis, and lung cancer, aiming to provide a systematic understanding of REE-induced lung toxicity and its pathological progression.

### 5.1 Interstitial lung disease (ILD)

Interstitial lung disease (ILD) encompasses a group of disorders characterized by diffuse inflammation and fibrosis of the alveolar walls, pulmonary lobular septa, and interstitial tissue, ultimately leading to impaired gas exchange and reduced lung compliance (54, 55). ILD subtypes include idiopathic pulmonary fibrosis, drug-induced pneumonitis, occupational pneumoconiosis, and exposure-related interstitial lung diseases (56). While classical causative agents are inorganic dusts such as asbestos, silica, and coal dust, exposure to REE nanoparticles in industrial, medical imaging, and military applications has emerged as a novel risk factor for ILD (1, 57).

Animal studies and case reports support a link between REE exposure and ILD. Ma et al. suggested that intratracheal instillation of cerium oxide nanoparticles (CeNPs) in rats led to alveolar wall thickening, inflammatory cell infiltration, fibroblast proliferation, and alveolar collapse—hallmarks consistent with ILD pathology (58). Co-exposure with diesel exhaust nanoparticles exacerbated interstitial collagen deposition and *α*-smooth muscle actin (α-SMA) expression, indicating synergistic pro-fibrotic potential (59, 60). In addition to experimental studies, clinical and postmortem findings corroborate these observations. Waring et al. reported a projectionist whose lung tissue exhibited high levels of Ce and La accumulation, accompanied by diffuse alveolar interstitial fibrosis, likely due to prolonged carbon arc lamp dust exposure (19). Comparable occupational cases have been reported in both industrial mining regions and military settings, where rare earth deposition is typically associated with alveolar wall thickening, interlobular septal sclerosis, and fibroblastic foci. The histopathological characteristics are consistent with nonspecific interstitial pneumonia (NSIP) or usual interstitial pneumonia (UIP) type ILD (61). These findings highlight that REE exposure often occurs as part of a broader environmental mixture, consistent with the exposome framework.

At the molecular level, Nd₂O₃ exposure activates the PI3K/Akt signaling pathway, upregulating inflammatory cytokines and fibrosis-related genes, and can modulate exosomal RNA expression to affect the tissue microenvironment (51). Zhang et al. (52) showed that Nd₂O₃ activates the lncRNA H19/miR-29a-3p/SNIP1/c-Myc axis, promoting abnormal proliferation of fibroblasts and extracellular matrix (ECM) deposition. In terms of immune regulation, studies have indicated that impaired autophagy in alveolar macrophages is a key factor in CeNPs-induced pulmonary fibrosis, whereas autophagy activation exerts a significant protective effect (61). Although large-scale epidemiological investigations are still lacking, existing case reports and observations in exposed populations provide preliminary epidemiological support for rare earth–induced ILD. For example, a population-based study from a rare earth mining area in southern China suggested that the levels of elements such as Ce and La in residents’ hair were significantly higher than those in the control area, and the incidence of ILD was markedly increased in the mining region, suggesting a potential link between environmental rare earth exposure and chronic pulmonary diseases (62).

In summary, REEs, particularly as nanoparticles, can deposit in alveolar regions via inhalation, potentially triggering inflammation, immune dysregulation, interstitial damage, and aberrant fibroblast activation, ultimately leading to ILD. Although evidence is largely from animal studies and case reports, pathological features are well-characterized, molecular mechanisms are being clarified, and epidemiological signals are emerging. REEs may be considered a potential emerging risk factor for ILD, warranting enhanced health surveillance and mechanistic research. However, most of the current evidence is derived from animal and *in vitro* studies, and its relevance to human exposure remains uncertain.

### 5.2 REE-associated pneumoconiosis

Pneumoconiosis comprises occupational lung diseases resulting from prolonged inhalation and deposition of inorganic dust, causing diffuse interstitial fibrosis. Common forms include silicosis, coal workers’ pneumoconiosis, and asbestosis (63). According to the 11th edition of the International Classification of Diseases (ICD-11) and the ILO classification criteria, pneumoconiosis is a typical occupational diffuse lung disease, prevalent in both industrialized and developing regions (64–66). Pathogenesis involves alveolar macrophage phagocytosis of dust, persistent oxidative stress, inflammatory mediator release, fibroblast activation, and ultimately alveolar structural destruction and irreversible lung function impairment (67–69).

In recent years, with the development of novel material industries, certain special inorganic particles have been found to induce non-traditional forms of pneumoconiosis, among which rare earth element–induced pneumoconiosis (REE-induced pneumoconiosis, REE-P) has attracted increasing attention (38, 70, 71). CeO₂ and La₂O₃ are widely used in metal smelting, electronics, and carbon arc lamp powders, often as ultrafine or nanoparticles that penetrate the lower respiratory tract and deposit in alveoli (72, 73). Early reports include Sabbioni et al.’s case of a photographic plate worker exposed to carbon arc lamp fumes for 46 years, whose lungs showed high Ce and La concentrations, supporting the diagnosis of REE-P (21, 71). Subsequently, Pairon et al. analyzed lung tissue from another patient with cerium oxide–induced pneumoconiosis using scanning electron microscopy (SEM) and energy-dispersive X-ray spectroscopy (EDX), confirming the deposition of cerium oxide particles within alveolar macrophages, accompanied by histological evidence of interstitial fibrosis and inflammatory cell infiltration (17). Jeremy et al. further supported the pathological basis of cerium-induced pneumoconiosis in a series of industrial exposure cases, noting that its fibrotic pattern may differ in certain aspects from classical silicosis, which is characterized by nodular fibrosis (74). Instead, cerium-associated pneumoconiosis more commonly presents with reticular fibrotic deposits and thickening of interlobular septa (75). Current evidence suggests that rare earth pneumoconiosis may exhibit certain pathological features that could differ from classical forms; however, these findings are not yet sufficient to establish specificity: first, alveolar macrophages often contain inclusions of rare earth metal particles, appearing as highly electron-dense granules or grayish-white crystals; second, some patients exhibit dendriform pulmonary ossification, a rare form of interstitial lung disease, indicating that rare earth particles may aberrantly activate osteogenic pathways—a phenomenon first reported by Song et al. in a patient with rare earth pneumoconiosis (76). Additional features include non-caseating granulomas, thickened pulmonary arterioles, alveolar disarray, and bronchial wall fibrosis, suggesting involvement of immune responses and local bone metabolism (77). Although several studies have suggested that REE-associated pneumoconiosis (REE-P) may exhibit features distinct from classical silicosis, such as more diffuse interstitial fibrosis, reticular patterns, and the presence of rare earth-containing particles within alveolar macrophages, these differences should be interpreted with caution. In many reported cases, workers were exposed to complex mixtures of dusts, including silica, carbon-based particles, and other metals. As a result, overlapping histopathological features—such as interstitial fibrosis, macrophage accumulation, and granulomatous reactions—are commonly observed, making it difficult to attribute the observed pathology solely to REEs.

It is noteworthy that REE-P is not limited to mining; occupational exposure exists in non-traditional settings such as film projection (19, 71). Due to long-term pulmonary retention of REE particles, toxic effects can accumulate, and disease progression may continue years after exposure cessation (16).

In summary, REE-associated pneumoconiosis is a relatively uncommon and diagnostically challenging occupational lung disease with complex pathological features. Importantly, it remains unclear whether REE-P represents a truly distinct clinicopathological entity or a variant of mixed-dust pneumoconiosis modified by the presence of REEs. While REE-P has been proposed as a distinct form of pneumoconiosis (21), current evidence remains insufficient to definitively establish its uniqueness, and its relationship with mixed-dust pneumoconiosis warrants further investigation. Given the frequent co-exposure to multiple inhaled particulates in occupational settings, it is plausible that REEs may act as modulators of pulmonary responses rather than sole causative agents. Diagnosis relies on occupational history, quantitative lung REE analysis, and histopathology. This perspective highlights the need for more rigorously designed studies. Future efforts should strengthen surveillance, early screening, molecular diagnostics, and mechanistic studies to inform effective occupational prevention strategies. Nevertheless, current evidence remains limited and largely based on case reports and small-scale occupational studies. The frequent co-exposure to multiple dusts further complicates the attribution of observed pathological features specifically to REEs. As such, the existing findings should be interpreted with caution, and the causal role of REEs in pneumoconiosis development requires further confirmation.

### 5.3 Lung cancer

Lung cancer remains the leading cause of cancer incidence and mortality worldwide, with multifactorial pathogenesis involving genetic susceptibility, smoking, occupational exposures, air pollution, and chronic chemical exposure. Widespread REE use in industry, medicine, and the military has raised concerns regarding their potential role in lung cancer development, making REE exposure an emerging focus in environmental toxicology and cancer epidemiology.

Multiple studies have supported that chronic REE accumulation in lung tissue is pronounced in populations living or working near REE mines or processing facilities (72, 73). This accumulation may alter the pulmonary microenvironment and has been hypothesized to contribute to carcinogenesis via DNA damage, gene mutations, and oxidative stress (78). Zhang et al. reported significant enrichment of 15 REEs, including Ce, La, and Nd, in tumor tissues compared to adjacent normal tissues, suggesting a possible association with the lung cancer microenvironment, although the biological significance of this finding remains to be further clarified (78).

Epidemiological studies support these findings. Residents in REE-rich regions of China (e.g., Jiangxi, Guangdong, Inner Mongolia) show elevated hair and blood REE levels and higher lung cancer incidence (79). In Xuanwei, Yunnan, Chen et al. found a positive correlation between REE content in local coal (Ce, Th) and age-standardized lung cancer mortality, suggesting that environmental REE exposure may be a potential regional risk factor (80). It is important to acknowledge that the interpretation of these epidemiological findings is subject to important limitations. In many studies, key confounding factors—particularly smoking history, age distribution, and co-exposure to other occupational or environmental carcinogens (e.g., silica dust, heavy metals, and air pollutants)—are not comprehensively adjusted for. As a result, the independent contribution of REE exposure to lung cancer risk remains difficult to ascertain.

At the molecular level, Liu et al. observed that REE exposure was associated with increased mutation frequencies in key driver genes (EGFR, TP53, KR), with stronger effects in female patients, suggesting potential sex-specific genotoxicity (81). Experimental studies indicate that Nd₂O₃ activates the lncRNA H19/miR-29a-3p/SNIP1/c-Myc axis, inducing fibroblast proliferation and extracellular matrix deposition, thereby creating a tumor-supportive microenvironment (52).

Cellular and animal studies further suggest dual biological effects. Mittal et al. reported that CeO₂ nanoparticles may cause DNA double-strand breaks, ROS bursts, and G₂/M cell cycle arrest in human lung epithelial cells, suggesting potential genotoxic effects *in vitro* and possible relevance to carcinogenic processes (53, 82). Conversely, Wang et al. found that a neodymium-containing complex (K₉(C₄H₄FN₂O₂)₂Nd(PW₁₁O₃₉)₂·25H₂O) have been shown to induced apoptosis in A549 cells, suggesting certain REE compounds may have anti-tumor activity (83).

The carcinogenic potential of radioactive REEs should also be noted. Cembe et al. reported that cerium chloride exhibited radioactive carcinogenicity in animals (84). Clinically, Y-90 radioembolization has been associated with radiation-induced pneumonitis and, in some cases, malignant pulmonary lesions, likely due to DNA damage and mutagenesis induced by radioactive REE isotopes in the pulmonary circulation (85, 86).

Although some REE nanomaterials (e.g., CeO₂ nanoparticles) suggest antioxidant and anti-tumor activity at low doses and have potential in drug delivery and imaging (87), long-term, low-dose, non-medical occupational or environmental exposure is more likely to exert adverse pulmonary effects, warranting careful consideration of carcinogenic risk in exposed populations.

In summary, Cumulative evidence from epidemiology, tissue analysis, cellular, and animal studies suggests that REEs may be associated with lung cancer–related biological processes through multiple mechanisms, although the evidence remains limited. However, the strength of evidence remains limited, and causality cannot be established due to insufficient control of confounding variables. While the data are still emerging, they highlight the need for long-term follow-up, cancer surveillance in exposed populations, and mechanistic research to clarify carcinogenic risk and inform effective preventive strategies. Moreover, inconsistencies across epidemiological findings and insufficient adjustment for key confounders, particularly smoking and co-exposure to other carcinogens, limit the interpretability of current evidence. Therefore, while mechanistic and observational data suggest potential associations, a definitive causal link between REE exposure and lung cancer remains to be established.

## 6 Molecular mechanisms of REE-induced lung toxicity

Taken together, current evidence suggests that the toxicological profiles of REEs cannot be generalized without considering their chemical speciation. Particle-associated REEs are characterized by localized pulmonary deposition, prolonged retention, and chronic inflammatory and fibrotic responses, whereas soluble REE forms are more likely to induce systemic effects through ionic interactions and disruption of cellular signaling pathways. This distinction is critical for bridging experimental findings with real-world exposure scenarios and for improving risk assessment strategies.

Building upon these toxicokinetic differences, the molecular mechanisms underlying REE-induced lung toxicity are complex and multifactorial. Although the mechanism of REE toxicity has been reported in several studies, research in this field still needs to be improved (88). Therefore, this review mainly explores the mechanism of REE toxicity from the following aspects: Oxidative Stress, Immune and Inflammatory Responses, and Calcium Homeostasis Dysregulation. However, these findings are primarily derived from experimental models and should be interpreted with caution when extrapolating to human populations.

### 6.1 Oxidative stress

Oxidative stress (OS) is widely considered a key mechanism mediating lung toxicity induced by REEs and their oxides (89, 90). OS occurs when the generation of reactive oxygen species (ROS) and reactive nitrogen species (RNS) exceeds the cellular antioxidant defense capacity, disrupting redox homeostasis and leading to lipid peroxidation, DNA damage, protein modification, and mitochondrial dysfunction, ultimately promoting apoptosis, necrosis, or aberrant repair (91, 92). Previous studies have shown that mineral dust, heavy metals, and nanoparticles have been shown to induce lung injury and fibrosis via oxidative stress, with rare earth elements exhibiting a particularly pronounced effect in this process.

*In vitro* studies have suggested that insoluble REE nanoparticles (e.g., CeO₂) primarily induce ROS production in bronchial epithelial Beas-2B cells and activate the p38–Nrf2 signaling pathway, upregulating antioxidant enzymes, such as heme oxygenase-1 (HO-1) and NAD(P)H quinone dehydrogenase 1 (NQO1). However, excessive exposure or unfavorable particle surface valence can overwhelm this defense, triggering pro-inflammatory cytokine release and exacerbating cellular injury (42). Environmental REE-containing dust has also been shown to induce ROS overproduction, DNA strand breaks, and micronucleus formation, indicating potential genotoxicity (93). Importantly, co-exposure with other nanoparticles, such as ZnO, may further amplify ROS-mediated cytotoxicity (94, 95).

Animal studies corroborate the pro-oxidative effects of REEs. Inhalation or intratracheal instillation of CeO₂ in mice significantly increased pulmonary malondialdehyde (MDA) levels, disrupted the activity of antioxidant enzymes (SOD, CAT, GPx), and induced neutrophil/macrophage infiltration and acute pneumonitis (96, 97). Chronic exposure led to particle accumulation, persistent ROS generation, and sustained inflammation, ultimately promoting fibrosis (97). Soluble REE salts, such as cerium chloride, similarly induced oxidative damage, highlighting the inherent pro-oxidant potential of REE ions (98).

**Figure 3:**
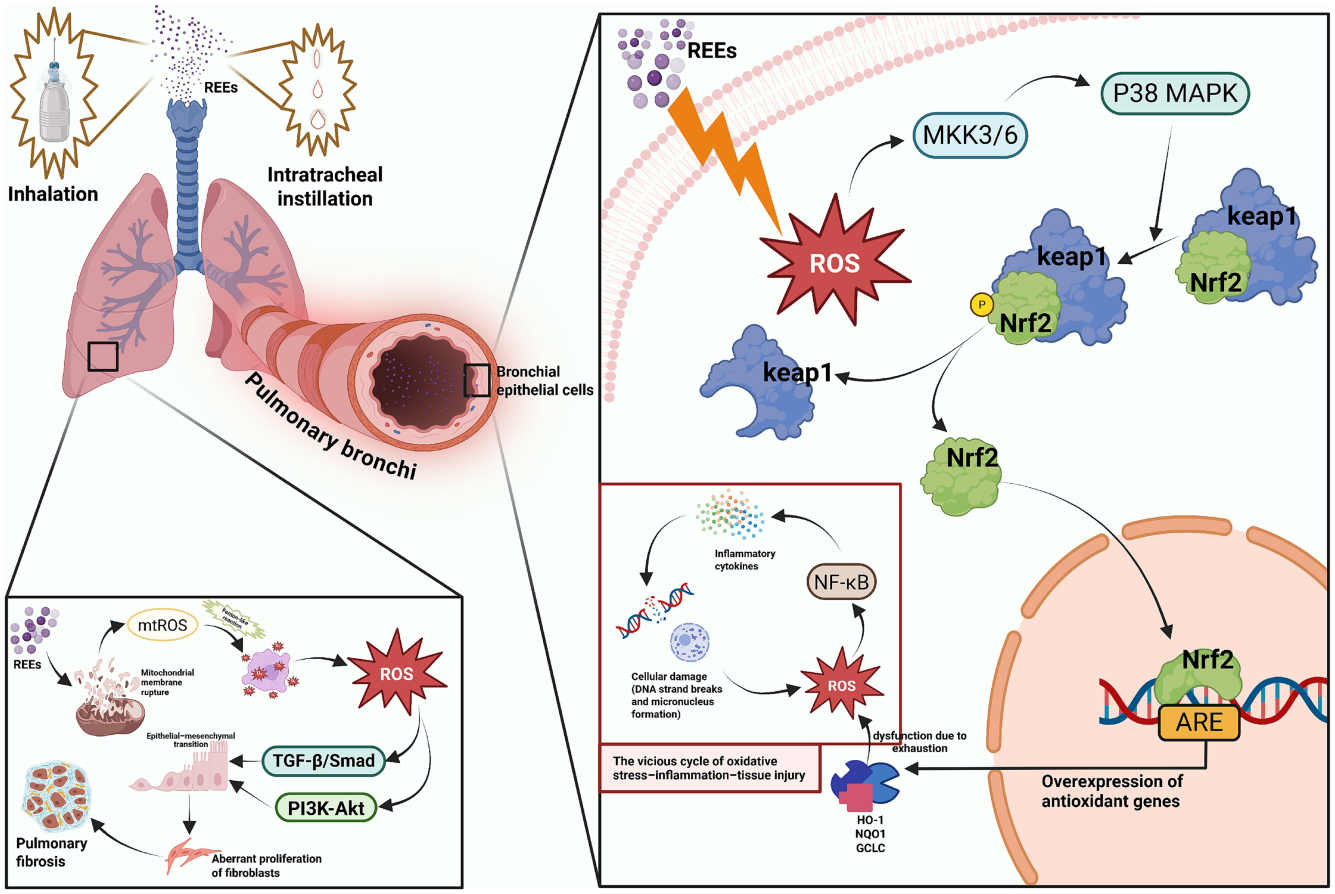
Oxidative stress-mediated molecular mechanisms underlying lung toxicity of REEs. Created with BioRender.com.

Mechanistic studies reveal multiple signaling pathways involved in REE-induced OS. The Nrf2/Keap1 axis is a critical regulator of antioxidant homeostasis, inducing expression of HO-1, NQO1 and GCLC. Under high-dose exposure, this pathway may be exhausted, exacerbating injury (99, 100). Simultaneously, MAPKs (p38, JNK, ERK) and NF-κB are overactivated by excessive ROS, promoting inflammatory cytokine release and NLRP3 inflammasome activation, forming a vicious “OS–inflammation–tissue injury” cycle (97, 101). REE particles can disrupt mitochondrial membrane potential, induce mtROS leakage, and catalyze Fenton-like reactions, further amplifying DNA damage and apoptosis (98). Chronic OS also activates TGF-*β*/Smad and PI3K-Akt pathways (102–104), promoting epithelial–mesenchymal transition (EMT) and fibroblast activation, thereby contributing to pulmonary fibrosis, as evidenced in Nd₂O₃ animal studies (97, 105) (see Figure 3). Notably, CeO₂ exhibits reversible Ce^3+^/Ce^4+^ valence cycling and surface oxygen vacancies, endowing it with nanozyme-like ROS-scavenging potential. At low doses or with proper surface modification, CeO₂ may exert antioxidant and anti-inflammatory effects, whereas high concentrations, defective surfaces, or co-exposures enhance pro-oxidant toxicity (94, 106). The dual effects of REEs are modulated by a combination of factors, including particle size, crystal phase, surface chemistry, exposure dose and duration, co-exposure scenarios, and individual host variability. Despite substantial experimental evidence supporting the role of oxidative stress, the extent to which these mechanisms operate under real-world human exposure conditions remains unclear. Differences in dose, particle properties, and exposure duration may significantly influence these outcomes.

These findings not only elucidate REE-induced lung injury mediated by ROS generation, antioxidant imbalance, and inflammatory cascades but also highlight occupational and public health risks. Workers in REE mines and surrounding residents may face OS-related damage and genotoxicity, warranting integration of biomarkers such as 8-OHdG, MDA, and GSH/GSSG ratio, alongside pulmonary function and imaging assessment in epidemiological and risk assessment studies (107). Subacute inhalation toxicology experiments have been proposed as a valuable approach to identify nanoparticle hazards, with implications for occupational health protection and novel material design.

### 6.2 Immune and inflammatory responses

Inflammatory response is among the earliest and most characteristic pathological mechanisms of REE-induced lung toxicity. Studies have shown that inhalation or intratracheal administration of REE oxides (e.g., CeO₂, Nd₂O₃, Yb₂O₃) results in pulmonary deposition and activation of innate immunity, triggering both acute and chronic inflammation (96, 108). This process involves disruption of the alveolar barrier, local inflammatory cell infiltration, and systemic inflammation mediated via cytokines and exosomal signaling (58).

#### 6.2.1 Innate immune activation and cytokine release

**Figure 4:**
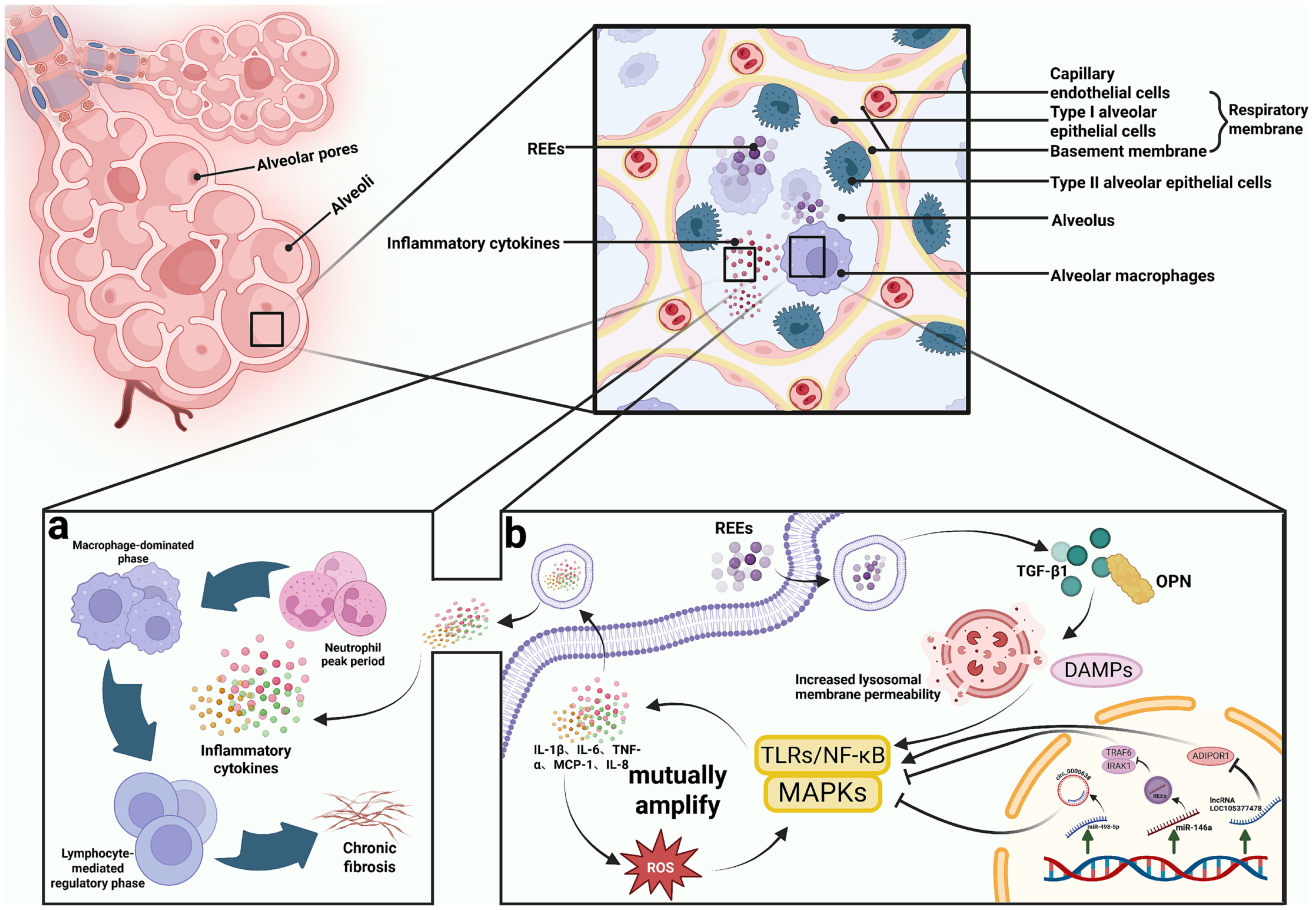
**(a)** The progression from acute inflammation to chronic fibrosis. **(b)** The reciprocal amplification between oxidative stress and inflammatory responses, along with the fine-tuning regulation by non-coding RNAs. Created with BioRender.com..

Upon entry into the lungs, REE particles are primarily phagocytosed by alveolar macrophages (AMs) and epithelial cells. AMs release fibrosis-associated cytokines such as TGF-β1 and osteopontin (OPN), increase lysosomal membrane permeability, and promote the release of damage-associated molecular patterns (DAMPs), activating TLRs/NF-κB, MAPKs, and other inflammatory signaling pathways (58, 109) (see Figure 4b). Animal studies have suggested significant upregulation of pro-inflammatory mediators, including IL-1β, IL-6, TNF-*α*, and MCP-1, accompanied by neutrophil recruitment and alveolar exudation (15, 101, 110). The pro-inflammatory effect persists even under well-dispersed CeO₂ conditions, highlighting the role of surface valence cycling (Ce^3+^/Ce^4+^) and chemical reactivity (111–113).

Under long-term exposure, rare earth particles such as Yb₂O₃ can accumulate in lung tissues and sustain inflammatory responses, leading to histopathological damage and an increased risk of fibrosis (114). Alterations in the gene expression profile of type II alveolar epithelial cells suggest a possible transition from acute inflammation to chronic fibrosis (115). Similar to the “inflammatory sequence” observed in radiation-induced lung injury, the immune response triggered by REE particles may sequentially undergo a “neutrophil peak phase—macrophage-dominant phase—lymphocyte-regulatory phase,” ultimately determining whether acute injury is repaired or progresses to chronic pathology (116) (see Figure 4a).

#### 6.2.2 Oxidative stress–inflammation crosstalk

REEs have been shown to induce ROS generation and impair endogenous antioxidant defenses, which further activates NF-κB/MAPKs pathways, promoting cytokine release and inflammatory cell infiltration. This forms a positive feedback loop—“ROS— signaling pathways— cytokines— inflammation amplification”—sustaining local and systemic inflammatory responses (117, 118) (see Figure 4b).

#### 6.2.3 Fine regulation by non-coding RNAs

At the molecular level, non-coding RNAs (ncRNAs) critically regulate REE-induced inflammation. Nd₂O₃ exposure in human bronchial epithelial (16HBE) cells triggers inflammation via lncRNA LOC105377478-mediated suppression of ADIPOR1, activating NF-κB and upregulating IL-6/IL-8 (119). Conversely, circ_0000638 exerts negative regulation through the miR-498-5p/NF-κB axis (120). CeO₂ NPs can also deliver miR-146a, which targets TRAF6 and IRAK1, attenuating pro-inflammatory cytokine production in mouse models of acute lung injury (121). Collectively, ncRNAs function as molecular switches, modulating inflammation intensity and duration (see Figure 4b).

Overall, REEs may contribute to lung inflammation by integrating innate immune activation, oxidative stress, and ncRNA-mediated fine regulation (99, 118). Acute exposure predominantly have been shown to induces transient inflammation, whereas chronic accumulation promotes progression toward fibrosis, highlighting inflammation as a central mechanism and potential target for intervention. Nevertheless, most of the current findings are based on controlled experimental settings, which may not fully capture the complexity of real-world exposure mixtures. Further studies integrating epidemiological and mechanistic data are needed to validate these pathways in human populations.

### 6.3 Calcium homeostasis dysregulation

Calcium ions (Ca^2+^), as a key intracellular second messenger, are involved in multiple processes in the respiratory system, including airway contraction and relaxation, inflammatory responses, maintenance of barrier function, and regulation of cell apoptosis (122). Pulmonary cells rely on tightly regulated mechanisms to maintain intracellular Ca^2+^ homeostasis, ensuring proper gas exchange and immune defense. Disruption, particularly Ca^2+^ overload, triggers physiological and biochemical disturbances, leading to cellular injury or death, which is highly relevant in lung diseases (123).

Previous studies have shown that REEs, due to their ionic radius and chemical similarity to Ca^2+^, can competitively bind to calcium-binding sites, perturb calcium channels, and interfere with downstream signaling, thereby disrupting Ca^2+^ homeostasis. In contrast, soluble REE salts (e.g., TbCl_3_, GdCl₃, LaCl₃) exert toxicity mainly through the release of free REE ions, which can directly interfere with Ca^2+^ channels and signaling pathways. For instance, terbium chloride accumulates in mouse lungs, significantly elevating free Ca^2+^ (124), while gadolinium chloride instillation in rat trachea induces pulmonary inflammation and tissue damage (125). Dysregulated extracellular Ca^2+^ promotes eosinophil transmigration and upregulates adhesion molecules CD11b/CD18, amplifying airway inflammation (126). REEs can impair mechanosensitive ion channels, altering endothelial Ca^2+^ oscillations and barrier integrity, leading to pulmonary edema and gas exchange deficits (127–129). Furthermore, lanthanum chloride inhibits neutrophil Ca^2+^ influx during chemotaxis, and gadolinium chloride impairs stretch-activated channels, disturbing epithelial–immune Ca^2+^ signaling and triggering inflammatory cascades (129, 130).

**Figure 5:**
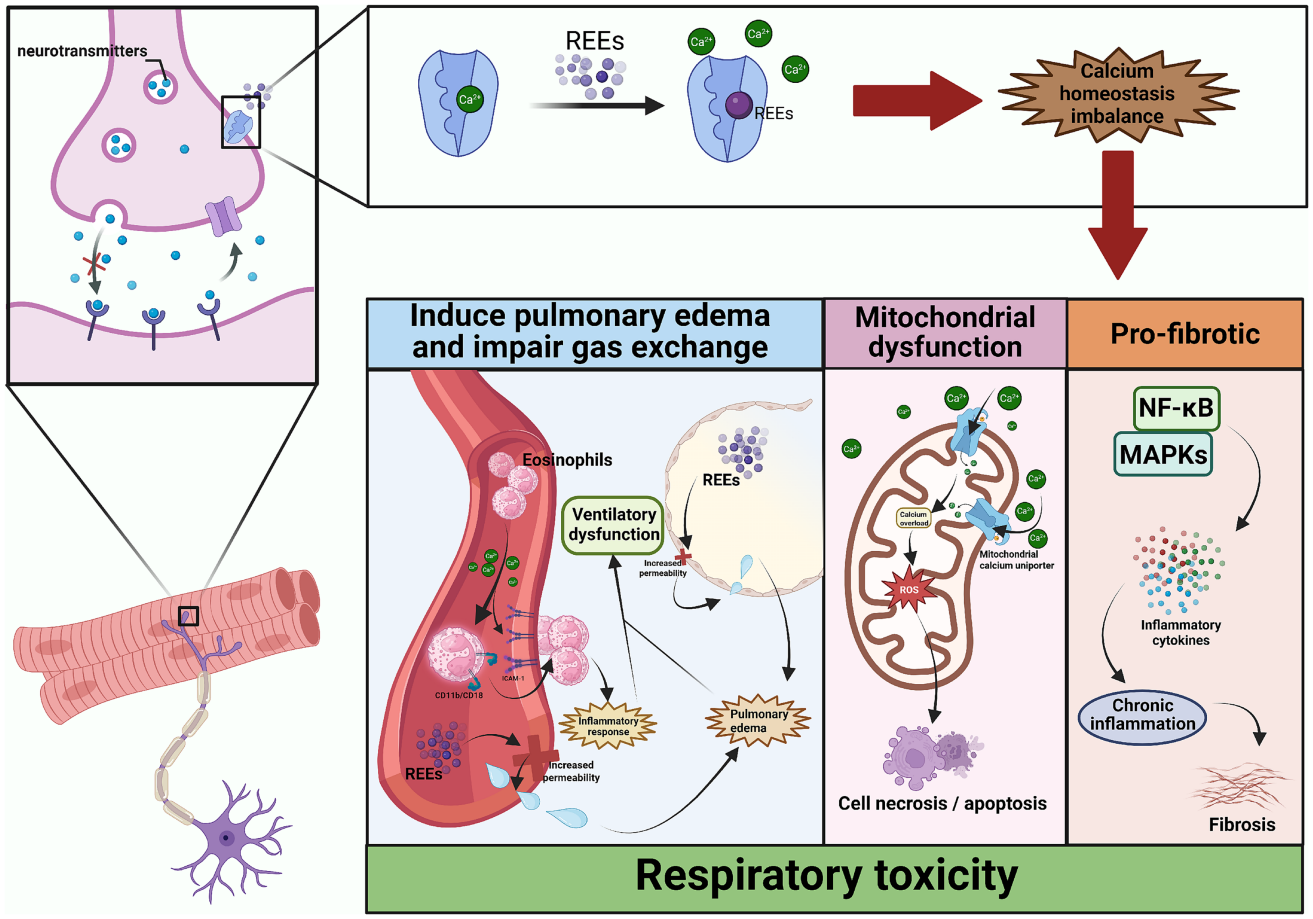
Molecular mechanisms of rare earth element-induced lung toxicity mediated by calcium homeostasis imbalance. Created with BioRender.com.

Critically, Ca^2+^ imbalance have been shown to induces mitochondrial dysfunction. Cytosolic Ca^2+^ overload drives excessive Ca^2+^ uptake via the mitochondrial calcium uniporter (MCU), causing mtROS overproduction, loss of membrane potential, and cell apoptosis/necrosis (131). Aberrant Ca^2+^ signaling also activates NF-κB and MAPK pathways, sustaining inflammation and promoting fibrosis (132, 133) (see Figure 5).

These results collectively indicate that REEs have been reported to disrupt pulmonary calcium homeostasis, potentially contributing to both acute injury and chronic inflammatory/fibrotic progression, providing new mechanistic insights into REE-induced lung toxicity. However, the current understanding of REE-induced calcium dysregulation is largely based on experimental studies, and its relevance to human respiratory disease remains to be clarified. Additional research is required to determine its contribution under realistic exposure conditions.

## 7 Conclusion

In recent years, rare earth elements (REEs) have emerged as a research hotspot in toxicology. However, the health hazards associated with REE exposure remain uncertain due to the synergistic toxicity of multiple elements in real-world environments, incomplete detection indicators, and individual differences in dynamic metabolism. This review focuses on the lung effects of REE inhalation, systematically summarizing exposure routes, major pulmonary diseases including interstitial lung disease, REE-induced pneumoconiosis and lung cancer, and molecular mechanisms such as oxidative stress, inflammatory responses and calcium homeostasis disruption. A common pathological spectrum—“inflammation–fibrosis–tumorigenesis”—was observed. Evidence suggests that REE particles may persistently accumulate in lung tissue, dysregulate multiple signaling pathways, and may contribute to structural and functional impairment of the respiratory system (18, 28). This process is modulated by particle physicochemical properties and exposure patterns. Firstly, the physicochemical characteristics of REE particles directly influence inflammatory responses. For instance, CeO₂ particles with different morphologies induce markedly different levels of inflammation (134), while immobilization on SiO₂ surfaces may confer anti-inflammatory and antioxidant effects (135). Secondly, the exposure mode is critical. Inhalation exposure more closely simulates occupational settings, and repeated exposure over 28 days can induce persistent low-grade inflammation and histopathological damage (101, 136, 137). Nevertheless, current evidence is largely derived from animal and *in vitro* models, with limited epidemiological data. Therefore, current evidence primarily supports associations rather than definitive causal relationships between REE exposure and respiratory outcomes, and should therefore be interpreted with caution.

It is noteworthy that REEs are not inherently “toxic” and may exhibit therapeutic potential under specific conditions. CeO₂ nanoparticles (CeO₂ NPs), owing to their reversible Ce^3+^/Ce^4+^ redox states and surface oxygen vacancies, can scavenge reactive oxygen species (ROS) at low doses, exhibiting antioxidant, anti-inflammatory, and cytoprotective effects (106, 111). Experimental studies suggest that CeO₂ NPs can mitigate oxidative stress and inflammation in ac confirmute lung injury and experimental pneumonia models and may regulate macrophage autophagy to confer protection against pulmonary fibrosis (61, 121, 135). Additionally, CeO₂ has been explored for drug delivery and cancer therapy, showing potential in synergistic chemotherapy and tumor progression inhibition in lung cancer research (53, 87). These findings suggest that REEs may exert dual effects—both toxic and therapeutic—depending on dose, form, exposure duration, and host factors. Future research should evaluate these aspects alongside their toxicological profiles.

## 8 Limitations of current evidence and research gaps

Despite growing interest in the respiratory toxicity of rare earth elements (REEs), several important limitations in the current body of evidence should be acknowledged.

First, epidemiological studies investigating the association between REE exposure and respiratory outcomes remain limited and are often subject to methodological constraints. Many studies are conducted in complex environmental or occupational settings (27), where co-exposure to other airborne pollutants (e.g., silica, particulate matter, or heavy metals) is common, making it difficult to isolate the specific effects of REEs. In addition, exposure assessment is frequently indirect, relying on environmental measurements or biological proxies (e.g., hair or blood concentrations) (29, 30), which may lead to exposure misclassification. Furthermore, potential confounding factors—including smoking (34), age, socioeconomic status, and pre-existing health conditions—are not always adequately controlled, thereby limiting causal inference. Thus, it is important to distinguish association from causation when interpreting the current body of evidence, particularly given the observational nature of most epidemiological studies and the presence of multiple environmental confounders.

Second, occupational studies of REE exposure often involve relatively small sample sizes and may lack appropriate control groups, reducing statistical power and generalizability. The long latency of chronic respiratory diseases further complicates the interpretation of exposure–response relationships.

Third, a substantial proportion of the available evidence is derived from animal experiments and *in vitro* studies. While these studies provide valuable mechanistic insights—such as oxidative stress (42), inflammatory signaling (96), and fibrosis-related pathways—their relevance to human health remains uncertain. Differences in exposure conditions, dosimetry, particle characteristics, and species-specific responses may limit direct extrapolation to real-world human exposures.

Finally, significant knowledge gaps persist regarding human-relevant exposure thresholds, long-term dose–response relationships, and validated biomarkers of early effect. The heterogeneity of REE species and their physicochemical forms further complicates risk assessment (see Table 2).

**Table 2:** Summary of key epidemiological and experimental studies on rare earth element exposure and respiratory health outcomes.

Section	Study	Study type	Population/model	REE exposure	Key findings	Limitations
Rare earth element exposure pathways	Dai et al. (29)	Epidemiological study	Residents in REE mining area	Environmental REE exposure (hair levels)	Elevated REE levels were associated with increased respiratory symptoms	Cross-sectional design; confounding factors
	Wang et al. (23)	Cross-sectional study	Occupational workers	Airborne REE dust	Exposure was associated with reduced pulmonary function indices	Cross-sectional design; lacks longitudinal follow-up
Interstitial Lung Disease (ILD)	Waring et al. (19)	Case report	Human (occupational exposure)	Long-term exposure to Ce and La dust	High accumulation of REEs in lung tissue was associated with diffuse fibrosis	Single case; co-exposure cannot be excluded
	Ma et al. (58)	Animal study	Mouse model	CeO₂ nanoparticles	Exposure was associated with increased expression of fibrosis markers	Mechanistic focus; unclear human relevance
REE-Associated Pneumoconiosis	Pairon et al. (16, 17)	Human study	Workers exposed to cerium oxide	Inhalation of CeO₂ particles	Demonstrated lung retention and biopersistence of cerium	Small sample size; mixed dust exposure
Lung Cancer	Chen et al. (80)	Ecological study	Regional population	REEs in coal combustion	Positive correlation between REE content and lung cancer rates	Ecological bias; smoking confounding
	Zhang et al. (78)	Observational study	Lung cancer patients	REE accumulation in tumor tissue	Higher REE levels in tumor vs. normal tissue	No causal inference; unclear mechanism
Molecular Mechanisms of REE-Induced lung Toxicity	Sisler et al. (89)	In vitro study	Human small airway epithelial cells	La₂O₃ nanoparticles	Suggested differential toxicity across metal oxides	Short-term exposure; controlled conditions
	Mittal and Pandey (82)	In vitro study	Lung adenocarcinoma (A549) cells	CeO₂ nanoparticles	Induced ROS generation and DNA damage	In vitro only; lacks in vivo validation

Taken together, these limitations highlight the need for well-designed longitudinal epidemiological studies, improved exposure assessment methods, and integrative approaches that bridge experimental findings with population-level evidence.

## 9 Quantitative exposure assessment and regulatory perspectives

Although increasing attention has been paid to the presence of rare earth elements (REEs) in ambient particulate matter and occupational settings, quantitative exposure assessment and risk characterization remain limited.

Currently, specific occupational exposure limits for most REEs are not well established. Only a limited number of elements have defined regulatory thresholds; for instance, the U.S. Occupational Safety and Health Administration (OSHA) has set a permissible exposure limit for yttrium at 1 mg/m^3^ as an 8-h time-weighted average (TWA). However, for the majority of REEs, exposure is regulated under general particulate matter or nuisance dust standards rather than element-specific guidelines. This lack of targeted regulatory benchmarks limits the ability to accurately assess exposure-related health risks and may not adequately reflect the unique toxicological properties of individual REEs.

From an international perspective, regulatory frameworks for REE exposure vary considerably. While some countries have proposed indicative exposure limits for certain lanthanides, globally harmonized standards are lacking. This inconsistency poses challenges for risk assessment and cross-study comparison.

Environmental monitoring studies have reported detectable concentrations of REEs in ambient air, particularly in regions with intensive mining, industrial processing, or electronic waste recycling. However, most available data are derived from studies conducted in China, with relatively limited information from other geographical regions, restricting the generalizability of current findings.

In addition, quantitative dose–response relationships between REE exposure and respiratory health outcomes remain poorly characterized. The lack of standardized exposure metrics, variability in particle size and chemical forms, and differences in exposure routes further complicate the interpretation of toxicological and epidemiological data.

Overall, there is a clear need for improved exposure assessment methods, establishment of evidence-based regulatory thresholds, and internationally coordinated monitoring efforts to better evaluate the health risks associated with REE exposure.

## 10 Challenges and perspective

Although REEs have become a hot spot in toxicology research in recent years, the hazards of REEs to the lung are still largely unknown. Looking ahead, future research should focus on: (1) Epidemiological evidence: conducting large-scale, long-term cohort studies integrating individual health records and high-resolution exposure monitoring to validate true health risks; (2) Safety thresholds and diagnostic criteria: establishing inhalation exposure limits and pneumoconiosis diagnostic standards in occupational and environmental populations, updating clinical and public health guidelines; (3) Innovative methodologies: applying multi-omics and single-cell approaches to decipher molecular networks, and employing airway organoids and “lung-immune system” organ-on-chip platforms to better simulate human exposure; (4) Interdisciplinary collaboration: strengthening integration across toxicology, materials science, epidemiology, and clinical medicine, promoting international data sharing; (5) Public health and policy translation: translating findings into occupational protection and environmental management strategies, including exposure monitoring, personal protective equipment, health surveillance, and evidence-based regulations. Future toxicological evaluation should explicitly consider dose–response relationships, physicochemical properties, and human-relevant exposure scenarios. What’s more, Future research should adopt an exposome-based approach to better capture the complexity of real-world exposures and their health effects.

In conclusion, studies on REE-induced lung toxicity provide critical insights into potential public health risks while also offering avenues for therapeutic applications. Establishing a bridge between mechanistic research and population health monitoring will facilitate risk assessment, development of diagnostic and treatment strategies, and provide a solid scientific foundation for public health policies and occupational safety standards.

## Glossary

8-OHdG: 8-Hydroxy-2′-deoxyguanosine
*α*-SMA: Alpha-Smooth Muscle Actin
AMs: Alveolar Macrophages
CAT: Catalase
CeNPs: Cerium Oxide Nanoparticles
circRNA: Circular RNA
DAMPs: Damage-Associated Molecular Patterns
ECM: Extracellular Matrix
EMT: Epithelial–Mesenchymal Transition
GCLC: Glutamate–Cysteine Ligase Catalytic Subunit
GPx: Glutathione Peroxidase
GSH/GSSG: Ratio of Reduced to Oxidized Glutathione
HO-1: Heme Oxygenase-1
HREEs: Heavy Rare Earth Elements
ILD: Interstitial Lung Disease
LREEs: Light Rare Earth Elements
lncRNA: Long Non-coding RNA
MAPKs: Mitogen-Activated Protein Kinases
MDA: Malondialdehyde
MCU: Mitochondrial Calcium Uniporter
miRNA: MicroRNA
MRI: Magnetic Resonance Imaging
mtROS: Mitochondrial Reactive Oxygen Species
ncRNAs: Non-coding RNAs
NF-κB: Nuclear Factor Kappa B
NLRP3: NOD-, LRR- and pyrin domain-containing protein 3 inflammasome
NQO1: NAD(P)H Quinone Dehydrogenase 1
NSIP: Nonspecific Interstitial Pneumonia
OS: Oxidative Stress
PI3K/Akt: Phosphoinositide 3-Kinase/Protein Kinase B signaling pathway
PM2.5: Particulate Matter ≤2.5 μm in diameter
REE-P: Rare Earth Element–Induced Pneumoconiosis
REEs: Rare Earth Elements
RNS: Reactive Nitrogen Species
ROS: Reactive Oxygen Species
TGF-*β*: Transforming Growth Factor Beta
TLRs: Toll-Like Receptors
UIP: Usual Interstitial Pneumonia
